# Collating Spirometry reference values in Asian children and Adolescents; puzzle out the reasons for variations

**DOI:** 10.12669/pjms.342.14162

**Published:** 2018

**Authors:** Sara. Sadiq, Syed Tousif Ahmed, Bina Fawad

**Affiliations:** 1 Dr. Sara Sadiq, M.Phil, MBBS. Department of Physiology, Ziauddin University, Karachi, Pakistan; 2 Dr. Syed Tousif Ahmed, M.Phil, MBBS. Department of Physiology, Ziauddin University, Karachi, Pakistan; 3 Dr. Bina Fawad, FCPS, MBBS. Department of Community Health Sciences, Ziauddin University, Karachi, Pakistan

**Keywords:** Determinants of spirometry, Spirometry reference ranges, Spirometry reference equation, Spirometry in Asian children, Spirometry reference values in children/ adolescents

## Abstract

Lung function tests are essential for the diagnosis and management of different respiratory tract diseases; among them the spirometry is the gold standard technique. The accurate diagnosis, management and monitoring require proper interpretation of the results which depends upon the availability of spirometry reference data for that particular region to differentiate the diseased condition from the normal ones. Multiple studies had been done to find out their own area specific reference ranges but it is still lacking. This need was fulfilled by the Global Lung Function Initiative (GLI) in 2012, which reported the first global spirometry equation for all of the age groups. But some of the studies reported difference among GLI reference range and the measured range for that particular region. So here is the review of the reference ranges among 35,603 Asian children and adolescents from the 32 studies done specifically in Asia. The aim was to compare them with the study done by GLI team, along with these, tried to rule out the causal factor that are responsible for the variations in the reference ranges among the children and adolescents of different population. The literature was searched by using Google scholar and PubMed during the month of March up to July 2017.

The review of all the articles published in Asia, specifically accounting for normal reference ranges in children and adolescent exhibit a wide variation among the reference ranges. This also suggest involvement of multiple modifiable and non-modifiable risk factors. So it’s necessary to update the reference ranges for spirometry and its prediction equation as well.

## INTRODUCTION

Respiratory diseases are the most common cause of morbidity and mortality worldwide, especially among children and adolescents. Lung function tests are essential for the diagnosis and management of different respiratory tract diseases; among them the spirometry is the gold standard technique. The accurate diagnosis, management and monitoring require proper interpretation of the results which depends upon the availability of spirometry reference data for that particular region to differentiate the diseased condition from the normal ones.^1^,^2^ These are some of the major factors that have an influence over the spirometry lung functions like age, sex, ethnicity and anthropometric variables.^3^-^5^ Some of the studies report the differences in spirometry lung function of white children and adolescents to those of Asians specifically the south Asians^5^-^8^, the reason for this difference may be the genetic factor^5^,^9^, it may be the anthropometric differences^10^-^15^, may be ethnicity^5^,^8^,^16^,^17^, or it may be the environmental factor like cultural factors, the socioeconomic status^6^,^18^-^24^ or the chemical exposure.^25^-^27^ So there is a need to keep up-to-date reference data.

According to the 2005 combined European Respiratory Society and American Thoracic Society (ERS/ATS) guidelines for spirometry^28^, there were very few publications for children as well as for adolescents in the Asian regions so it’s very difficult to generate a regression equation and to generalized it. This need was accomplished by the Global Lung Function Initiative (GLI) in 2012 which reported the first global spirometry equation for all of the age groups. This was multi-ethnic equation i.e. for 5 different ethnic groups, including Caucasian (for White), African-American (for Black), North-East Asian (for North China, Korea), South-East Asian (for South China, Thailand, Taiwan) and Other (for mixed ethnic origin).^29^

However, some of the studies reported difference among GLI reference range and the measured range for that particular region^12^,^13^,^25^ while most of the studies didn’t compare their reference values. So the objective of this study was to review the reference ranges among Asian children and adolescents and to compare them with the study done by GLI team. The other objective of this study was to review the causal factor that is responsible for the variations in the reference ranges among the children and adolescents of different population.

## METHODS

The literature was searched by using Google scholar and PubMed during the month of March up to July 2017. The keywords used for the literature search was “Spirometry reference ranges in children of Asia”, “Spirometry reference ranges in adolescent of Asia”, “Spirometry in children and adolescents”, “Pulmonary function test reference ranges in children” and “Pulmonary function test reference ranges in adolescents”, “Pulmonary function test in children and adolescents”, “Lung function test in children and adolescents”, along with these all particular country name was also mentioned in search engine. The search result showed of total 4611 articles including all the 48 countries of Asia. Those articles that mentioned the spirometry ranges for different diseases like asthma, cystic fibrosis, acute respiratory diseases, COPD, linked to obesity, sickle cell disease, SLE, idiopathic juvenile arthritis, chronic cough, allergic rhinitis, recurrent pneumonia, bronchiolitis obliterans, bronchiectasis were excluded that’s about 4551.

Out of 60 articles, those studies which were either done in pre-term low birth weight children or done in pre-school children or those articles which were not available in English were also excluded. This review included only those articles which published spirometry reference ranges for Asian children and adolescents of age range between 5-18 years, that is around 32 article. The bibliography of selected articles was also checked out for relevant articles.

The independent sample T-test was used to find out the association of gender with the spirometry lung volumes while correlation model was applied for the association of age and anthropometric variables with the spirometry variables. On the other hand, the mean calculated values were compares with the GLI reference range by using ANOVA. P-value <0.05 were considered as statistically significant. Analyses were performed using SPSS version 20.

## RESULTS

The entire Asian data for age (specifically children and adolescents), height, arm span, weight, FVC, FEV_1_, FEF_25-75%_, PEFR, FEV_1_/FVC ratio were comprehended for 18,113 males (that is 2021 from Central Asia, 4162 from East Asia, 2339 from Southeast Asia, 2332 from South Asia, 7259 from West Asia) and 17,490 females (that is 2024from Central Asia, 4089 from East Asia, 2435 from Southeast Asia, 1722 from South Asia, 7220 from West Asia).

### Comparison with GLI 2012 Reference Equation

Most of the studies had been done in Caucasians or had a very small number of samples so affects the application of a gold standard technique. This problem had been solved by GLI 2012 spirometry reference equation which was very effective as they mentioned the different reference equations for every ethnic groups.^29^

However, after establishing these reference equations some of the studies reported variation in the reference range, this may be due to the fact that Quanjer used the data that were established several years ago. Like one of the study done in Chinese Han population compared their reference ranges to that of the GLI reference range, given specifically for Asian population and they reported the higher spirometry lung volumes in their population while the GLI showed lower range.^12^ On the other hand one of the study showed lower spirometry lung volumes as compared to the reference range given by GLI team^25^ and this was supported by a Gypsy study that also reported lower FVC and FEV_1_ volumes in both boys and girls as compare to their predicted one by Quanjer’s team.^30^ But still there are some studies that supports the use of GLI reference equation in their population.^13^,^31^

**Fig.1:**
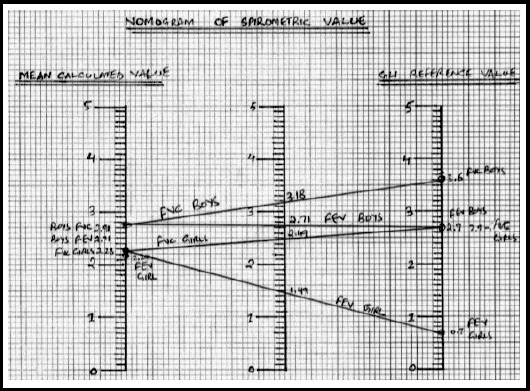
Nomograms of Spirometry reference values.

At the end of the study Quanjer mentioned that the spirometry data provided by South-Asians to the GLI team, was not comparable with mean results and to derive reliable reference equations. So for Asia, they only mentioned the values specifically for the individuals living in North East Asia and South East Asia.^25^,^29^ Table-I shows Statistically significant difference when the GLI ranges were compared with the mean calculated values obtained from all the studies done among the children and adolescents of Asian population. These results confirmed the mean calculated spirometry lung volumes were lower than the reference range given by GLI team. On the other hand, Nomograms of the Spirometry GLI reference values with mean calculated values has been made to ease the clinical practice as mentioned in Fig.1.

**Table-I:** Comparison among GLI reference values and the mean calculated reference values.

Lung Function Parameters	GLI Reference Values	Mean Calculated Values	p-value
**FVC**			
Boys	3.6 ± 0.19	2.71 ± 0.79	0.020
Girls	2.1 ± 0.23	2.28 ± 0.63	
**FEV**_1_			
Boys	2.7 ± 0.13	2.71 ± 0.65	0.027
Girls	2.07 ± 0.19	2.20 ± 0.66	
**FEF**_25-75%_			
Boys	3.2 ± 0.67	3.53 ± 0.58	0.010
Girls	7.7 ± 0.70	3.23 ± 0.61	

### Major determinants for variations among Spirometry reference ranges

***1. Age:*** Age is not just a number, Studies reported that in children and adolescents age has a positive correlation with the spirometry variables.^32^ As it increases the strength of reference equation because in children and adolescents as the age increases, the power of respiratory muscles also increases along with it especially the intercostal muscles as well as there is increase in elastic recoil of lung.^11^ As the study by Tahera et al. revealed an increase in FVC, FEV_1_ and PEFR within the age range of 9 to 16 years.^19^

However, literature review revealed no significant correlation among the age and spirometry lung volumes as shown in Table-II.

**Table-II:** Correlation of age with spirometry lung volumes.

	Df	Mean Square	F	P- Value
Age Boys	4	7.015	0.260	0.900
	18	26.966		
	22			
Age Girls	4	5.881	0.251	0.905
	18	23.444		
	22			
FVC Boys	4	0.585	0.910	0.477
	20	0.643		
	24			
FVC Girls	4	0.211	0.472	0.756
	20	0.447		
	24			
FEV_1_ Boys	4	1108.637	2.055	0.127
	19	539.388		
	23			
FEV_1_Girls	4	0.294	0.924	0.471
	19	0.318		
	23			

***2. Sex:*** Gender also shows a very important difference as many studies reported higher lung volumes in males as compare to that in females. One of the study gave possible explanation that is about difference in lung growth pattern so Alfrayh et al. suggested that there should be different reference equation for both the sexes.^11^ A study done in India supported this finding and according to that the reference equation is affected more by the height in males.^7^

Our review result showed no significant correlation of gender with the spirometry lung function as mentioned in Table-III.

**Table-III:** Correlation of Gender with spirometry parameters.

Gender		Mean	Std. Deviation	P- Value	95% Confidence Interval of the Difference	
					Lower	Upper
FVC	Boys	2.4176	0.79586	0.114	-0.08221	0.73821
	Girls	2.0896	0.63815	0.115	-0.08271	0.73871
FEV1	Boys	2.1782	0.65501	0.343	-0.19696	0.55512
	Girls	1.9992	0.66741	0.343	-0.19697	0.55513

***3. Ethnicity:*** Ethnicity is a well-established fact for the variation in spirometry volumes. As the literature reported difference in ranges among ethnic groups like Indian study showed lower values as compare to their age mates of American blacks or European origin and they are supposed to take additional steps for formulating the regression equation.^33^

***4. Anthropometric variables:*** One of the study shows there is slight decrease in spirometry lung volumes in Indians children as compared to their age mates having same ancestry living in different countries.^25^ Most of the studies reported that height always have a linear correlation with the spirometry lung volumes but weight has no any significant correlation.^32^,^34^ When look over the south-east Asia, an Indian study showed greater influence of height in males while in females the weight is predominant^7^, this finding is also supported by a study done in china that’s results manifest a strong correlation of weight with lung volumes than the age in females^12^, so these suggests a positive correlation of both height and weight among all anthropometric variables with the lung volumes.^35^ On the other hand one of the study manifest a significantly positive correlation of FVC, FEV_1_ and PEFR with the weight but negative correlation of FEV_1_% with the body surface area.^19^

A Chinese study noted the fact that as the height increases, the lung size increases along with it, this in turn leads to increase in lung capacities and volumes.^17^ This is the linear type of relationship among height and lung volumes up to the age of 12 in boys and 10 in girls but during puberty it becomes non-linear type.^13^ Literature review revealed a positive correlation of both height and weight with lung volumes, they considered height as an independent variable with high value of coefficient of correlation.^33^,^36^ Because of this reason one of the study exclude age and weight from the prediction equation by keeping height as the main variable but this created bias, this problem was resolved by using height and age both as an independent variable in the equation while weight was dropped out.^12^ In our study we found no significant correlation of height as well as weight with the lung function test in either sex. The detailed results are shown in Table-IV.

**Table-IV:** Correlation of Anthropometric variables with calculated pulmonary function Parameters.

Variables	Mean	FVC	P- Value	FEV_1_	P- Value
Height Boys	146.02	2.48 ± 0.79	0.373	2.31 ± 0.66	0.387
Weight Boys	34.16		0.894		0.928
Height Girls	145.28	2.2 ± 0.64	0.394	2.02 ± 0.67	0.233
Weight Girls	33.78		0.130		0.101

***5. Socioeconomic Status:*** Socioeconomic conditions and nutritional status are the two variables that can’t be neglected as they are the important ones for the lung development and growth. Sonnapa et al. did a comparison study among the children of different socioeconomic status, having same ethnic origin, the study concluded that those individual who have the poor socioeconomic status from rural areas having lower lung functions as compare to the individuals who are from the well stable families. So the study suggested to consider the socioeconomic status as an important risk factor along with other variables.^25^

The literature review manifest that in China spirometry related studies done in early 90’s, after that there was a progression in economic development that raised the standard of living so Ya-Nan et al. revised the spirometry reference ranges. He took the children born in between 1997-2003, his results reported increase in spirometry reference ranges.^17^ This result is also supported by a Japanese study whose results showed that life style modification and improvement in health and nutritional status have positive effect over the lung volumes.^13^ He-Q et al. divided their study participants into two groups, the comparison showed that the group with high intake of leafy vegetables, fresh fruits and milk had higher FEV_1_ than the other group while FVC difference was insignificant among both groups.^24^

All these findings gave strength to include the socioeconomic status as an important determinant for spirometry reference ranges.

***6. Biomass smoke Exposure:*** This is a well-established fact that women and children from developing countries are exposed to indoor air pollutants produced from cooking, baking and biomass fuels like woods, agricultural crop residues, and charcoal mainly in rural areas while families from urban areas are exposed to kerosene and liquid petroleum gas (LPG).^37^ None of the study had been done to find out the direct association between biomass smoke exposure and the lung volumes in children or adolescents. Very few studies reported indirect association of biomass smoke exposure with structural and functional disruption in respiratory system that increases the prevalence of asthma and COPD.^37^,^38^

## CONCLUSION

The review of all the articles published in Asia, specifically accounting for normal reference ranges in children and adolescent exhibit a wide variation among the reference ranges given by GLI team and the calculated one. The suggested reason for these variations may be the ethnicity, socioeconomic status or the biomass smoke exposure while no significant association of age, sex, height and weight were found. It’s necessary to update the reference ranges for spirometry and its prediction equation as well.

### Authors’ Contribution

**SS** has designed the study, was involved in acquisition, analysis, and interpretation of data.

**SS and BF** did statistical analysis.

**SS and STA** drafted the manuscript and revised it critically for important intellectual content.
